# Automation of literature screening for meta-analyses in the field of psychology: an evaluation of two text-mining approaches

**DOI:** 10.1186/s13643-026-03275-x

**Published:** 2026-08-05

**Authors:** Dennis Gloukhman, Caroline Lade, Ulrich S. Tran

**Affiliations:** 1 Department of Cognition, Emotion, and Methods in Psychology, Faculty of Psychology, University of Vienna, Vienna, Austria; 2 Pfizer Pharma GmbH, Berlin, Germany

**Keywords:** Literature screening, Article selection, Text-mining, Automation, Meta-analysis

## Abstract

**Background:**

The process of consolidating research through meta-analyses is time-consuming and costly. Using text-mining approaches to (semi-)automate the step of screening and selecting articles eligible for inclusion might potentially save hours of workload and costs. A lack of evaluative research on the use of text-mining for literature screening prevents the widespread adoption of text-mining approaches among the psychology research community. This study explores the possibilities of applying text-mining approaches to facilitate or (semi-)automate screening articles for inclusion in psychology meta-analyses.

**Methods:**

The performances of two publicly accessible text-mining approaches were evaluated and compared against three recent meta-analyses from different subfields of psychology.

**Results:**

Averaged over 10 text-mining runs per meta-analysis, across the three meta-analyses the approaches achieved recall performances between 62.5% and 99.3%, specificities between 60.1% and 92.7%, and saved between 54.0% and 92.0% of screening workload. Higher recall was observed when more articles were screened manually in the text-mining process.

**Conclusions:**

These findings suggest that text-mining approaches can substantially reduce screening workload while maintaining high recall. These efficiency gains come with a trade-off: the more workload is saved, the lower the recall performance. Text-mining can therefore serve as a valuable tool for the initial identification of potentially eligible studies or as a secondary screening aid, but the risk of missing relevant studies should be mitigated by retaining an appropriate amount of manual screening.

**Supplementary Information:**

The online version contains supplementary material available at https://doi.org/10.1186/s13643-026-03275-x.

## Background

With an ever-growing literature, the value of performing a meta-analysis (MA) increases. However, conducting an MA is a time-consuming and costly process [1]. A big part of MAs is to screen all articles retrieved from databases to determine their eligibility for inclusion in the MA [2]. Specifically, this step of literature screening can take up to weeks and months depending on the numbers of articles that need to be screened [3]. Therefore, new approaches to manage this workload are needed. To accelerate this process and thereby reduce hours of workload and associated costs, recent advances in automating literature screening have been made using text-mining (TM) algorithms. That is, TM extracts information and patterns from huge amounts of texts and documents in a computerized process. Used for literature screening, TM may reduce the amount of time required to create an MA decisively [4, 5]. However, TM approaches also tend to miss eligible studies [4, 5], which makes their potential benefits doubtful. Also, their relative novelty and the lack of technical skills and knowledge in the research community may hinder their application. Currently, evaluations of the applicability and performance of TM approaches on literature screening are lacking in the social behavioral sciences and psychology. This may hinder the application of TM in the workflow of MAs in these research fields. This study aims to evaluate if TM approaches can be beneficially used to automate literature screening for MAs in the field of psychology in order to minimize costs and the time spent on literature screening.

There are many promising TM approaches for literature screening (e.g., [4–12]). The unifying feature of these approaches lies in the underlying task of text classification to assign articles to predefined classes [9] based on which articles will be included in or excluded from an MA. A class could be any arbitrarily chosen category, such as the use of the same medical intervention. Text classification could be based on titles, abstracts, full article texts, and other information that is commonly reviewed during literature screening.

Recently, large language models (LLMs) have further advanced the capabilities of TM. LLMs constitute a class of TM tools trained on vast amounts of data that can generate coherent texts and dialogues and show strong potential for improving text classification performance. However, LLMs are costly to develop and deploy, require specialized hardware, and their classification decisions are often difficult to interpret. Most importantly, the use of LLMs may lack reproducibility, as different runs can yield different results, and access to models is often restricted [13]. Reproducibility is a key requirement for transparent and reproducible research, and the lack of reproducibility severely impacts the quality of research.

In contrast, conventional TM methods are computationally efficient, can be run inexpensively on standard hardware, and typically offer greater transparency, properties that are particularly important in resource-constrained MA research where methodological transparency is valuable. Moreover, traditional TM relies on explicit algorithmic and statistical procedures that allow direct inspection of features and decision criteria, thereby facilitating reproducibility. Rather than being outperformed by LLM-based approaches, traditional TM continues to provide a robust and lightweight analytical framework that can function as a standalone screening aid or complement LLMs in hybrid analytical pipelines for literature screening in MAs.

Using TM for selecting eligible studies in standard literature screening processes has not become common practice yet. Researchers first skim all titles and abstracts of articles retrieved from a database search manually. If they suspect an article to meet the pre-defined inclusion criteria, they note it down for later full-text assessment, to check whether it meets all criteria. While it is often only possible during full-text assessments to determine whether a study fully meets the inclusion criteria, it requires substantially more time compared to assessing titles or abstracts alone. The whole process is usually conducted with the help of digital tools or platforms, which sometimes even have integrated TM functionalities. TM approaches could accelerate the selection process. However, their inability to detect all relevant studies and the lack of control by the researcher over the study selection are seen as key obstacles to broaden acceptance among the research community [4]. The completeness of the selected literature in the screening step is decisive for the subsequent scientific quality of the MA. Accordingly, using TM for study selection may have a strong impact on the MA’s quality.

However, as the completeness of selected literature is also subject to human error (e.g., diverging interpretation of subject or inclusion criteria, inattentive article screening) too [14], TM approaches can contribute large value by pre-selecting relevant articles prior to manual screening or by constituting as second screener. That is, TM could be used to sort or prioritize articles, that are more likely to be eligible for inclusion, for manual screening [4], as is standard for digital tools that support the screening process. Finding eligible articles earlier in the process allows for parallelizing subsequent steps within the research team. When using TM as a second screener, it has the great advantage of one human researcher retaining control over the final study selection and process while substituting the second human screener [4]. TM approaches could thus potentially cut the required screening time in half. While a computerized approach for screening does not guarantee the completeness of the selected studies, it certainly reduces the likelihood of human errors through computerized counterchecking and could help save funding resources through increasing the human screener’s efficiency.

Nevertheless, evidence and trust gaps remain for the general research community to adopt TM as a standard practice in the process of MA creation [4, 15]. Acceptability is currently hindered specifically by the identification of the best approaches for literature screening, limited ease of use, scarce comparative research on the same MAs or gold-standard datasets across multiple disciplines, and the lack of replications of evaluation [4]. Thereby, the generalizability of TM application across different research fields might be a key aspect to increase TM adoption. For instance, O’Mara-Eves et al. [4] reported a lack of studies evaluating TM approaches for literature screening in the social behavioral sciences. Most extant TM approaches were evaluated with MAs from the clinical or pharmaceutical fields. Yet, these types of studies tend to differ from studies in social behavioral sciences or psychology in various ways (e.g., more standardized terms, randomized controlled trials, etc.). The differing characteristics of different fields might impact the performance of TM approaches. Thus, performance evaluations from one (sub)field might have limited generalizability to another (sub)field of research. Thus, TM approaches should be evaluated with MAs from different research fields or subfields. More such comparative research is needed to increase acceptability across research fields.

Another key aspect to hindering acceptability among the common research community, is the lack of research to identify the best TM approach for literature screening. One large difficulty in the identification is constituted by sensible TM performance metrics. Most published TM approaches use metrics like recall, precision, or accuracy to facilitate performance comparability. However, even when using the same TM approach in different MAs, TM performance can result in high scores in one MA, but lower scores in another MA. That may be because of differing characteristics of the MAs, for instance, different population inclusion criteria, or variability of terminology used for a topic within a field. Therefore, to obtain more accurate performance evaluations, it is necessary to use the same MA for the comparison of different TM approaches, and more than one MA for the evaluation of individual approaches, using the same performance metrics in all cases. Currently, such comparative work is scarce [4, 5]. In sum, for better comparability, different TM approaches should be applied to the same MAs, but from different research fields or subfields.

The present study aimed to close some of the evidence and trust gaps of TM application for automated literature screening presented above by comparing the performance of two different TM approaches on three MAs from three different subfields of psychology. This allowed identifying performance differences between different approaches for the same MAs across different subfields of a single discipline. As evidence for the application of TM approaches to MAs from the social behavioral sciences is currently missing, we chose psychology as a diverse field of research, with many distinct subfields and interdisciplinary approaches. TM approaches were chosen for their reported good performance, applicability, and ease of implementation (e.g., open-source code in the coding language R). The nature of the present study was mostly exploratory, but in part also educational, as it guides the reader through, and explains, the required steps and workflows of the two TM approaches in screening the literature for an MA. The outcome of this study further informs on whether TM facilitates literature screening in the subfields of psychology and whether this helps to reduce the time required for study selection while still retrieving most of the eligible studies in MAs in psychology.

## Methods

Two publicly accessible TM approaches [10, 11] for literature screening were applied to three MAs within three subfields of psychology [16–18]. To evaluate the TM approaches’ performance, the number of retrieved articles eligible for inclusion were contrasted with the original number of articles that were manually selected by the original authors of the MAs.

Both TM approaches have been applied in their source papers in clinical research, but not in the field of psychology. Both TM approaches were reported to retrieve high proportions of eligible articles in their source papers (88%–100%) but lack sufficient re-application and evaluations on MAs from other fields to determine the generalizability of their performances. Both TM approaches were implemented in the coding language R [19], which is widely used in psychology and might hence be accepted and adopted by the research community of psychology for literature screening. The R code of both TM algorithms was available via open access. All three MAs chosen for this study were recently published, are well documented, and cover different subfields of psychology: Agostini and van Zomeren [16] from social and political psychology; Everaert et al. [17] from cognitive and clinical psychology; and Roberts et al. [18] from cognitive and neuroscience.

### TM approaches

**Fig. 1:**
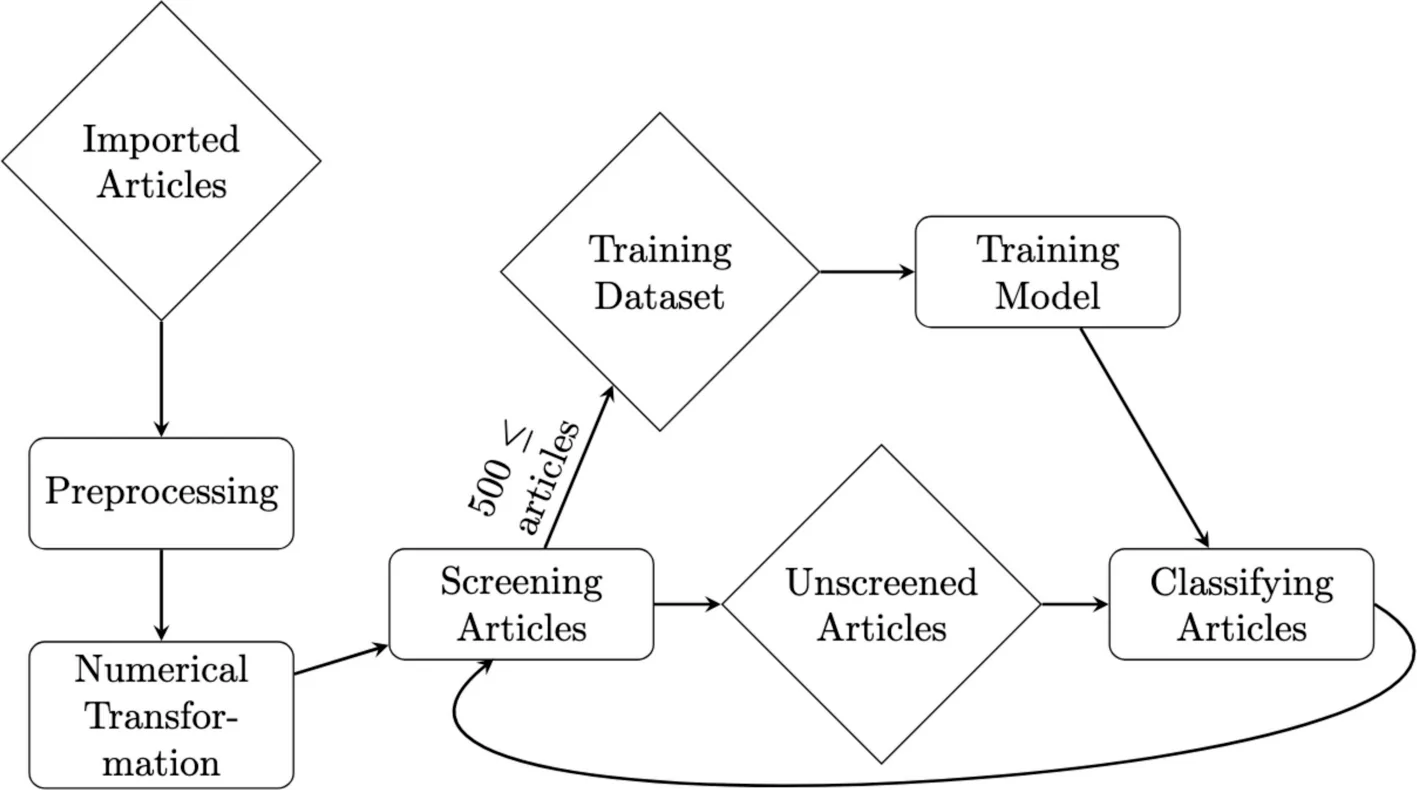
Illustration of the general workflow for Pham et al. [10] and Popoff et al. [11], as adapted in the present study (see main text for details)

The two TM approaches by Pham et al. [10] and Popoff et al. [11] were selected based on the above-mentioned criteria among citations that referenced the well-known review by O’Mara-Eves et al. [4] on TM approaches for literature screening. Neither had been applied to non-clinical MAs. While Popoff et al. [11] was applied in five MAs of clinical randomized controlled trials on diseases like cancer, obesity, or psoriasis, Pham et al. [10] was evaluated in one MA on type-1-diabetes and in one scoping review on knowledge synthesis methods. Likewise, there has not been any comparative evaluation between Pham et al. [10] and Popoff et al. [11] on the same MAs so far. A simplified general workflow of both approaches is illustrated in Fig. 1. For full details, see Pham et al. [10] and Popoff et al. [11] and their published code files. All code and the datasets generated and/or analyzed during the current study are available in the OSF repository, https://osf.io/kb9mn/.

### Importing articles

Once all articles for literature screening have been gathered, titles, abstracts, and arbitrary unique IDs of all studies can be passed to both TM approaches in any structured text formats or tables that are readable by computers (see Supplementary Material for technical details). While full text can also serve as input, potentially improving TM performance [12], it is often not freely accessible and subject to subscription fees and additional time is required for locating and retrieving each study. While Pham et al. [10] used titles and abstracts as input for their TM approach, Popoff et al. [11] used only abstracts. Consequently, the present study relied solely on titles and abstracts as input for TM for both approaches, just as in Pham et al. [10].

### Preprocessing & numerical transformation

After importing the articles, inputs are pre-processed to remove any meaningless information and patterns. To this end, duplicate studies or copyright information from publishers or databases are removed. Lastly, the text inputs need to be transformed into a document feature matrix (DFM), a numeric representation of the text inputs in a tabular form, where rows represent individual articles, columns depict arbitrarily chosen features of the article’s text, and values reflect the frequency of the respective feature. In other words, text inputs are transformed to frequency values. Popoff’s et al. [11] TM approach transforms it to one DFM with features representing the frequency of individual words, irrespective of word category information. Pham’s et al. [10] TM approach transforms the text input into three different DFMs. The first DFM contained words as features that appear in direct proximity of each other within any given sentence, restricted up to three nouns or verbs (words of other word categories were removed). The second DFM used 300 topics as features, with frequency values representing how often each topic appeared in the articles. The third DFM was constructed using word embeddings, a technique that captures contexts by focusing on semantic similarity and relationships between words across the documents.

### Screening articles to create a training dataset

To allow for adequate comparison between the two TM approaches, the training dataset for both TM approaches was created following Pham’s et al. [10] approach in this study. In the approach of Popoff et al. [11], random sampling was then applied to this training dataset (described in more detail below). To start off the screening process, for the application of Pham’s et al. [10] TM approach, five studies were randomly selected per MA from the respective list of included studies to serve as seed articles. Seed articles constitute the base for pattern recognition of inclusion criteria across all articles. Based on these seed articles, a training dataset was created. The number of articles included into a training dataset is arbitrarily set. Pham et al. [10] recommended reviewing between 300 and 600 abstracts to achieve reliable results. However, if the algorithm identifies more or fewer articles than the preset threshold used to stop the training dataset creation, the final number of included articles will differ accordingly.

In our case we adopted Pham’s et al. [10] threshold of 600 articles. Researchers are then required to actively screen all articles of the training dataset and to provide feedback whether to include or exclude an article (the so-called active learning workflow).

In contrast, for application of Popoff’s et al. [11] TM approach, information on which articles to include into the training dataset is assumed to exist from the start. The training dataset was thus partitioned through random sampling (75% training/25% test; as in the original approach of Popoff et al. [11]) of all articles screened in the original MAs, with a sampling ratio of 1:1 (include: exclude) in the present study. This implies an MA update scenario, where a major portion of the literature was already screened in advance. It is not applicable to the scenario of creating entirely new MAs, since decisions on which articles to include are missing and no training dataset could be created without available decisions.

### Training model

Next, the model tries to detect patterns and characteristics to accurately divide the articles in the training dataset into two classes, included or excluded articles, by determining rules and steps. For example, an easy pattern could be a frequent usage of certain terms, or term combinations, in the articles’ abstracts. By generalizing the learned patterns from the training dataset to new unscreened articles, the model is then able to infer which articles are likely eligible for inclusion. Pham et al. [10] employs a random forest algorithm, whereas Popoff et al. [11] employs a support vector machine. A random forest is an ensemble of consecutive random decisions to split the data based on its features. The output is a collection of consecutive rules which produced the best classification performance. A support vector machine creates a model which also classifies the articles into ‘included’ or ‘excluded’ classes. However, instead of consecutive feature-splitting decisions, it uses a mathematical hyperplane to divide the dataset into the two classes based on the features.

### Classifying articles

After training the model, the TM approach is ready to automatically classify all remaining articles not included in the training dataset for eligibility of inclusion into the MA. In the approach of Pham et al. [10], only articles which are classified as included are subsequently manually screened and upon the decision of final inclusion added to the previous training dataset. The training of the model, the screening of the included classifications, and the updating of the training dataset is iteratively repeated until the model does not find any new eligible articles during the subsequent screening. Pham et al. [10] reported that their approach terminated after 5 iterations which resulted in manual screening of 37%–45% of all studies in their original evaluations. With 5 iterations they found all eligible articles in the first MA and missed 6 eligible articles in the second MA, which corresponded to an error rate of 1.5%.

In contrast, the approach of Popoff et al. [11] produces two probabilities, one to include articles, one to exclude articles. Probabilities above a threshold of 0.9 to either include or exclude lead to a classification by the algorithm. Articles with probabilities below this threshold need to be manually screened and are not classified. Popoff et al. [11] reported that their original approach missed up to 6% of eligible studies for selection.

In the present study, we adapted the original workflow of Popoff et al. [11] in three ways. First, all articles with probabilities below the threshold were assumed to belong to the excluded class and were not screened; only articles classified as included were manually screened to check whether they meet the inclusion criteria. Second, the threshold of inclusion was set to either 0.8 or 0.6 to potentially increase the algorithm’s recall. A lower threshold might decrease the potential time saved due to more articles classified as included, but in turn might increase the recall. An initial trial with a threshold of 0.9 left almost all articles as unclassified or as excluded. Third, the iterative repetition (training the model, classifying, and screening the articles until no new eligible articles could be found) from the approach of Pham et al. [10] was applied in the approach of Popoff et al. [11] as well. These adaptations were introduced to ensure better comparability to Pham et al. [10], but also to save screening time, as the original approach by Popoff et al. [11] initially left almost all articles unclassified, necessitating the manual screening of all articles. Table 1 provides an overview of deviations from the original TM approaches.

**Table 1:** Overview of deviations to the original TM approaches

	Pham et al. [10]	Deviation	Popoff et al. [11]	Deviation	Reasoning for deviation
Input to TM	Abstracts & titles	None	Abstracts	Abstracts & titles	To provide a fair comparison between approaches
Training dataset creation	Part of approach to create training dataset	None	MA update scenario; implies training dataset already exists	Using the dataset according to the approach of Pham et al. [10]	To provide a fair comparison between approaches and due to restriction to MA update scenario in original approach
Threshold for dataset creation	600	None	75% of all articles are used for training	Using the dataset according to the approach of Pham et al. [10]	To provide a fair comparison between approaches
Classification probability threshold	NA	NA	.900	.800 and .600	Using a threshold of .900 in an initial trial run on the Everaert et al. [16] dataset resulted in almost the entire dataset to remain unclassified. Values were determined before evaluations were conducted
Iterative application/active learning	Applied iteratively until no new eligible articles are found	None	Only single iteration	Applied iteratively until no new eligible articles are found	To provide a fair comparison between approaches
Definite classification	Classifies articles either as included or excluded	None	If classification probability is below preset threshold, article remains unclassified	Unclassified articles are treated as excluded	To provide a fair comparison between approaches & potentially more time savings that could justify the TM application

*NA* Not applicable

### MAs for evaluating the two TM approaches

Three MAs were selected based on the following six criteria: First, selected MAs were required to be from different subfields of psychology. Second, MAs were required to have been published in a scientific journal with a journal impact factor (JIF) > 5, using JIFs as an imperfect, but workable proxy to ensure the overall relevance and quality of the MA. Third, the literature screening process was sufficiently documented. To this end, only MAs using the PRISMA reporting guideline [20] were considered to ensure sufficient documentation. Fourth, MAs were required to include databases for the literature search that were frequently used in psychology (e.g., PsycInfo, PubMed, or Web of Science). Fifth, MAs were required to have at least some of the used databases in common. Sixth and finally, as it is recommended by Pham et al. [10] to have at least 5000 articles for the TM approach to perform well during literature screening, the present study aspired to have some variation in the initial number of studies to screen per MA to assess its leniency. Therefore, at least one MA was required to have screened more than 5000 articles, one around 5000, and one fewer than 5000 articles.

The three MAs were found in an advanced search on 10 February 2023 on the APA PsycNet database, with filters in methodology set to Meta Analysis, source journal *to Psychological Bulletin*, with no other search string entered. *Psychological Bulletin* was considered as the source journal as it publishes high-quality peer-reviewed articles from many subfields of psychology. The following three MAs from the field of clinical, social, and cognitive psychology, respectively, were identified and chosen as test cases for the present study: Agostini and van Zomeren [16], Everaert et al. [17], and Roberts et al. [18]. Table 2 provides an overview of their characteristics. All three MAs included at the end almost 150 studies or more, indicating well researched topics.

**Table 2:** Overview of meta-analyses selected from *Psychological Bulletin* for evaluating the two TM approaches

	Everaert et al. [17]	Agostini and van Zomeren [16]	Roberts et al. [18]
Subfield	Clinical & cognitive psychology	Social, cultural, & political psychology	Cognitive, neuro & clinical psychology
Topics	Memory, depression	Motives for collective action	Enactment effect
Goals	1. Assessing effect size of explicit long term memory bias 2. Identifying moderators of effect size	1. Replicating results with new data 2. Synthesizing a cross-cultural model, which integrates multiple motives for collective action	1. Creating a detailed catalogue of enactment articles 2. Summarizing the efficacy of study and study factors 3. Synthetizing behavioral with neuroimaging and patient studies
Databases	PsycInfo, Embase, Pubmed, Proquest, citation searching	PsycInfo, Web of Science, e-mail correspondence	PsycInfo, Pubmed, Scopus, Web of Science, Proquest, citation searching, e-mail correspondence
Articles	Until August, 2021	June, 2007 – September, 2018	1981 – September, 2020
Screened	6665	8567	2285
Full text assessed	518	456	413
Included	154	231	148

For Everaert et al. [17], the literature search was replicated as described in the paper, for Agostini and van Zomeren [16] as well as Roberts et al. [18] it was re-created from publicly shared files, containing the results of the original authors’ own literature search. While the reconstruction of datasets based on article inclusion and exclusion decisions may introduce limitations in terms of data completeness and labeling accuracy affect absolute estimates of recall, no fully annotated original psychology meta-analyses were available for the purposes of this study. To mitigate these risks, we cross-validated our reconstructed datasets against available supplementary information in the original MAs and conducted consistency checks, and we now provide the reconstructed datasets with their inclusion/exclusion labels as open materials to support transparency and reuse. Moreover, reconstructing evaluation datasets or creating entirely new ones is a common practice in the evaluating TM for MAs research community (e.g., [6, 8, 21]).

Datasets used as input for the TM approaches consisted of 8228 articles for Everaert et al. [17], 7432 articles for Agostini and van Zomeren [16], and 1339 articles for Roberts et al. [18]. Table 3 displays the detailed literature re-creation and the final composition of the MA datasets. Deviations between the counts of originally identified articles by the original authors of the MAs and recreated literature searches in the present study can be explained by the unstructured or incomplete shared data files, untraceable additional articles from alternative data sources like other researchers and institutions, and unpublished datasets.

**Table 3:** Composition of meta-analyses used as the ground truth to evaluate TM approaches

	Everaert et al. [17]		Agostini and van Zomeren [16]		Roberts et al. [18]	
	Original	Replicated	Original	Replicated	Original	Replicated
Literature search						
Total Articles	12,490	15,131	8587^a^	8560	2455^a^	1338
Database	12,490	-	8567	-	2285	-
Proquest	204	687	-	-	used^a^	-
Web of Science	4313	5115	used^a^	-	used^a^	-
PsycInfo	3034	4141	used^a^	-	used^a^	-
Pubmed	2197	2803	-	-	used^a^	-
Embase	2729	2385	-	-	-	-
Scopus	-	-	-	-	used^a^	-
Unspecified	-	-	8567	-	2285	-
Other	13	-	20	-	170	-
Unpublished	17	-	8	-	72	-
Duplicates	5825	6903	not stated	1137	946	-
Final dataset composition						
Left to Screen	6665	8228	8587	7432	1338	1338
Excludes	6522	8094	8336	7241	1193	1200
Includes	143^b^	134	231	191	148	139

Database searches were fully replicated only for Everaert et al. [17]. For the other two meta-analyses, replications were based on publicly accessible dataset documentations found on https://osf.io/v7bwk/ and https://osf.io/f4ymv/. Discrepancies in numbers might stem from unstructured or incomplete data, not stated additional sources, and unpublished datasets. Retrieving these missing articles was considered beyond the scope of this study. The final replicated datasets were used to evaluate whether the TM approaches would find the included articles

^a^ Authors reported only total number of articles found in all databases

^b^ Authors reported 154 datasets from 143 articles

It was expected that the performance of both TM approaches would be best for Everaert et al. [17], because it had the most coherent terminology among the three MAs and provided more training opportunity for the TM approaches due to its large set of articles. Performance for Agostini and van Zomeren’s [16] and Roberts’ et al. [18] MAs was presumed worse due to their broad concepts and less coherent terminology across their subfields. TM application on Roberts et al. [18] was presumed to lead to the worst performance as the number of articles in the dataset was lower than the recommended 5000 by Pham et al. [10].

### Performance metrics

The performances of the automated MA literature screening were evaluated against the results of the manual literature screening of the reconstructed MAs with respect to the completeness of included eligible articles identified via TM application.

The TM approaches were also evaluated based on established metrics: recall (i.e., sensitivity), specificity, precision, accuracy, and F1 scores. Recall measures the percentage of true positives from all positives, that is, the percentage of studies selected by the algorithm. A high recall reflects a high percentage of eligible articles identified, whereas a low recall implies that a high percentage of eligible articles was missed. Specificity indicates the percentage of excluded studies which are true negatives. A specificity of 100% means that all ineligible studies were correctly excluded, thereby reducing the manual screening workload to eligible studies only. Precision denotes the percentage of true positives from all studies selected by the respective TM approach. Accuracy measures the percentage of correct classifications from all classifications, considering not only the true positives but also the true negatives. F1 is the harmonic mean of precision and recall and balances the importance of selecting all eligible articles, but not to the expense of selecting too many ineligible articles (false positives). In other words, whereas an eligible article in the training dataset increased recall, an ineligible article decreased precision, since all articles in the training dataset were considered classified as included and needed to be screened.

Finally, based on Wallace’s et al. [22] estimates, potential time saved was defined as 30 s on a lower bound, and 2 min on an upper bound for calculations on average manual screening time that was potentially saved (i.e., average time saved per article not screened). The potential time saved does not account for time used for other necessary steps, when applying TM: TM setup (e.g., article import, text pre-processing, text transformation, or dataset creation), computational, or code executing time. However, these depend on highly individual factors, such as the number of databases used, the technical expertise of the researcher, and the size of the source literature for the MA. Potential time saved thus quantifies only the likely increase in efficiency when TMs are already set up for the screening process.

Recall, specificity, and potential time saved (or the percentage of articles that were not screened) were considered the most important metrics for evaluating the applicability, and potential success of TM for literature screening. These metrics best reflect the core goal: identifying all eligible articles, while saving screening workload at the same time. To avoid result dependence on the five seed articles required to start the TM approaches, ten independent runs were conducted for each approach. The performance metrics reported in Table 4 therefore represent averages of the summary statistics across ten runs each. For each run all reported metrics were calculated using both the training and the test datasets. This was necessary as – due to the iterative nature of the TM approaches – focusing on the metrics during one final iteration, or on the test dataset alone, would not adequately reflect the performance of the TM approaches.

**Table 4:** Performance of Pham et al. [10] and Popoff et al. [11]

Metric	Replicated meta-analysis								
	Everaert et al. [17]			Agostini and van Zomeren [16]			Roberts et al. [18]		
	Pham et al. [10]	Popoff et al. [11]		Pham et al. [10]	Popoff et al. [11]		Pham et al. [10]	Popoff et al. [11]	
Threshold	NA	.8 ^a^	.6 ^b^	NA	.8 ^a^	.6 ^b^	NA	.8 ^a^	.6 ^b^
Classification metrics									
Recall	0.725	0.625	0.761	0.788	0.666	0.805	0.993	0.955	0.963
Accuracy	0.871	0.922	0.904	0.845	0.92	0.913	0.642	0.682	0.682
Specificity	0.873	0.927	0.906	0.846	0.926	0.916	0.601	0.65	0.65
Precision	0.087	0.124	0.119	0.119	0.193	0.201	0.224	0.24	0.242
F1	0.155	0.207	0.206	0.207	0.299	0.322	0.365	0.385	0.386
Article screening metrics									
Screened articles	1123 (58)	677 (45)	859 (100)	1264 (65)	660 (33)	763 (40)	617 (2)	552 (5)	554 (5)
Screened in %	14	8	10	17	9	10	46	41	41
Saved time in %	86	92	90	83	91	90	54	59	59
Saved time in hours ^c^	59–395	63–420	61–409	51–342	56–376	56–370	Jun-40	Jul-44	Jul-44
Found includes	97 (2)	84 (5)	102 (8)	151 (3)	127 (5)	154 (7)	138 (0)	133 (1)	134 (1)
Includes training	79 (3)	79 (3)	79 (3)	111 (5)	111 (5)	111 (5)	133 (1)	133 (1)	133 (1)
Includes approach	19 (4)	5 (3)	24 (6)	39 (6)	16 (3)	42 (4)	5 (1)	0 (0)	1 (1)
Includes in original	134	134	134	191	191	191	139	139	139
Missed includes	37	50	32	41	64	37	1	6	5
Missed in %	28	38	24	21	33	20	1	5	4

Numbers are averages 10 runs with 5 draws of random seed studies. All values in the classification metrics section have a *SD* ≤.035. For article screening metrics, *SD*s are in parentheses

*NA* Not applicable

^a^ Articles with probability threshold >.8 selected for inclusion

^b^ Articles with probability threshold >.6 selected for inclusion

^c^ Estimated range with lower bound on the left-hand side and upper bound on the right-hand side

## Results

Table 4 shows the TM approaches’ performance. For more detailed results containing the confidence intervals, see the Supplementary Materials. The two TM approaches achieved recalls between 62.5% to 99.3%, specificity rates between 60.1% to 92.7%, and saved between 54.0% to 90.0% of manual screening time. Both TM approaches achieved the best performance on Roberts’ et al. [18] MA with a recall rate as high as 99.3%, 95.5%, and 96.3%. Recall was lower for the other two MAs, with 72.5%, 62.5%, and 76.1% for Everaert et al. [17] and 78.8%, 66.6%, and 80.5% for Agostini and van Zomeren [16]. While recall was high for Roberts et al. [18], the relative percentage of manually screened articles was also higher for Roberts et al. [18] than for the other two MAs (> 40% vs. < 20%) leading to the lowest percentage of saved time (54%) for Roberts et al. [18], compared to the other two MAs. Overall, applying Pham et al. [10] and Popoff et al. [11] with two different thresholds showed that across all three MAs Popoff et al. [11] slightly outperformed Pham et al. [10], when using a threshold of 0.6 and the same training dataset.

Above and beyond the performance metrics, we noted variations in computation time reflected by the differing numbers of total articles in the respective MAs. For computations to pre-process the data, to transform the texts into numerical data, for the TM algorithm to suggest similar articles to the seed articles, and to subsequently create a training dataset according to Pham et al. [10] took approximately 13 h for Everaert et al. [17], 10 h for Agostini and van Zomeren [16], and 1 h for Roberts et al. [18]. The code scripts of both approaches were easily adapted and reapplied to work on the present MAs. For further details on the adaptation of code script for TM application to the three MAs, see the Supplementary Materials. All code and the datasets generated and/or analysed during the current study are available in the OSF repository, https://osf.io/kb9mn/.

## Discussion

The general aim of the present study was to examine whether TM approaches could facilitate literature screening in MAs in the field of psychology in reducing time and workload in selecting eligible studies. Further, to contribute to identifying the best TM approaches, two TM approaches were applied to three MAs from three different subfields of psychology. Performance was contrasted to the article selection of the original studies. The results showed that while the two TM approaches succeeded in saving time, they also missed articles eligible for inclusion. For two of the replicated MAs, the two best working approaches missed 19%–24% of all eligible articles, while saving 83%–92% of manual screening time. For the third MA, the two TM approaches missed only 1%–4% of eligible articles, but saved only 54%–59% of manual screening time. Contrary to our expectations, TM recall performance was best for Roberts’ et al. [18] MA and worst for Everaert’s et al. [17] MA. However, in terms of potential time saved, TM application was more beneficial for literature screening of Everaert’s et al. [17] than Roberts’ et al. [18] MA. Performance appeared to depend on the relative proportion of all articles that were manually screened for the training dataset. Screening an absolute number of at least 600 articles resulted in a much higher proportion of initially screened articles in the creation of the training dataset for Roberts et al. [18] than for the other two MAs. Overall, while TM approaches substantially reduced the required workload, they also missed a considerable number of eligible articles. This suggests that TM should not be used as a standalone screening method but can either serve as valuable aid for human screeners for pre-selecting relevant articles prior to manual screening or serve as a second screener for MAs in psychology.

Both TM approaches succeeded in selecting eligible articles for inclusion across different subfields of psychology, reflected in an average recall of 80% reaching up to 99%. Consistently, both approaches achieved their highest average recall (97%) for Roberts et al. [18], followed by Agostini and van Zomeren [16] (75%), and the lowest average recall (70%) for Everaert et al. [16]. The high recall performance for Roberts et al. [18] was likely attributable to the comparatively smaller number of articles and higher percentage of manually screened articles to create a training dataset from among all three MAs. It was surprising to see the TM approaches perform better on Agostini and van Zomeren [16] than on Everaert et al. [17]. Agostini and van Zomeren [16] was the MA with the most diverse concepts and theories in its research question and highest relative lack of uniform vocabulary, compared to the more clinically oriented MA of Everaert et al. [17]. Thus, TM appeared to be applicable to literature screening in the diverse subfields of psychology, even for MAs with quite diverse study characteristics.

Both TM approaches also performed comparatively well, and achieved similar recalls, when applied to the same MA. Popoff et al. [11] achieved higher average recalls than Pham et al. [10] except for Roberts et al. [18]. Though, the comparison was inconclusive since Popoff et al. [11] was adapted in the present paper and diverged from the recommended classification threshold of 0.9 which led to the TM performing consistently worse than Pham’s et al. [10] TM. To conclusively infer which of the two approaches performs superior, further research needs to be conducted. Currently, both approaches appear to be equally applicable overall to the literature screening of MAs in psychology.

Both TM approaches also notably reduced manual screening time, saving between 54 to 90% of the workload, while correctly excluding ineligible articles with specificity rates between 60.1% and 92.7%. Assuming an average screening time of 30 s to 2 min per article, this translates to potential time savings of approximately 59 h to 395 h for those specific MAs assessed here. Importantly, estimates for workload save do not account for TM setup, model computation, or code execution time, as these highly depend on individual differences in researchers’ expertise.

Though the TM approaches reduced time for manual screening, this came at the cost of missing more eligible articles compared to the original MA human-based literature screening. But the more articles were screened manually during TM application, the fewer eligible articles were missed. For instance, applying Pham et al. [10] to the literature screening of Roberts et al. [18] saved only 54% of screening workload, but also only missed 1% of eligible articles. The number of screened articles depends on both the classification decisions made by the TM approach and the arbitrarily chosen number of articles that had to be included in the training dataset. Hence, TM approaches have the advantage of potentially saving workload and making the MA creation process more efficient, but as a trade-off they might miss eligible articles.

Achieved average recalls were lower compared to the papers that published the two TM approaches. Likely, the crucial factor for lower recalls in the present study was the lower percentage of manually screened articles to create the training datasets, relative to the volume of unscreened articles. Pham et al. [10] reported to have manually screened 37% and 45% of articles to create a training dataset, for Popoff et al. [11] this number even reached 75%. In contrast, in the present study, for Everaert et al. [17] and Agostini and van Zomeren [16] only 10% to 17% of all articles were manually screened, for Roberts et al. [18], it was between 41 to 46% of articles – similarly high proportions of screened articles compared to the source papers of the TM approaches. The difference in the proportion of screened manually articles resulted from the arbitrary threshold of 600 articles which Pham et al. [10] recommended to screen to create a training dataset. For Roberts et al. [18] that 600 articles constituted a higher proportion of the overall number of articles compared to the other two MAs. Likewise, Roberts et al. [18] was the only MA for which the TM approaches achieved similarly high recalls as in the TM source papers. Consequently, in addition to an absolute minimum, such as 600 articles as the threshold, it appears to be advantageous to use a certain *percentage* relative to the number of articles in the dataset (e.g., 40% or 45% of all articles) to achieve high recalls. In turn, if increasing screening to such extent would reliably turn out recall performance as high as for Roberts et al. [18], that is 99%, TM approaches can potentially save over half of the workload needed for literature screening and thus increase efficiency of human screeners conducting MA.

However, the recall performance obtained in this study for two of the MAs may be viewed as below the level generally expected for using TM as a reliable tool to support screening. Since missing eligible articles severely impacts the quality of MAs, the general goal would be to find all (or nearly all) eligible articles and thus to maximize the recall of the TM approaches. While no minimum threshold for acceptable recall values was established in the literature, presumably recalls above 95% could be deemed acceptable in return for potential workload savings and thus making the process of MA creation more time efficient. However, both TM approaches investigated in the present study passed this threshold only for Roberts et al. [18], but not the other two MAs. Yet, using TMs as a second screener or as a prioritization system, which suggests articles that are more likely to be eligible to be screened earlier in the process [4], effectively maintains human control over the screening process while enabling other research team members to progress earlier to later stages of MA creation, consequently enhancing the overall efficiency of the workflow. By making a strong effort to avoid missing eligible studies, both strategies can help ensure the overall quality of MAs and offer promising, time-efficient approaches for the psychological research community to deploy TM. Applying TM approaches requires, at least, an intermediate level of programming and familiarity with TM techniques. Without these skills and knowledge, the application and adaptation of the TM approach to new MAs might be challenging for researchers. As demonstrated in this study, re-using and adapting the code files for the application of TM approaches to MAs in psychology was feasible and facilitated by the published and well-commented code files of the utilized TM approaches. Nevertheless, without prior programming skills and knowledge about TM, understanding each step of the code and solving technical issues (see online supplementary material) might be challenging.

To overcome this starting barrier, it is advisable for MA researchers to focus on one particular TM approach and to practice it before conducting research with it. Pham et al. [10] argued that learning to apply their approach without prior knowledge would be possible within three months. With prior knowledge and having applied their script once, adapting their code script to conduct a new MA might be feasible within a few minutes. Another option would be to opt for serviced ready-to-use software solutions. Both fee-based solutions (e.g., EPPI-Reviewer, DistillerSR, rayyan), and free solutions (e.g., Abstrackr, ASReview) exist. While code file solutions give more control to the researcher and do not incur additional costs, software solutions with graphical user interfaces might be easier to use for the research community at large without requiring intermediate programming knowledge. Thus, TM approaches are re-usable and adaptable to new MAs, but for researchers without the required skills learning to apply the approach might be initially cumbersome.

Noteworthy, both TM approaches were blind to the actual article inclusion criteria. They rather exploited the fact that articles included in an MA tend to be very similar in their research topics and the words used and deviate from one and another only in various study parameters. Thus, in one way or another, both TM approaches investigated in the present study estimated the similarity of articles or word patterns, compared to eligible articles in the training datasets, and selected articles with high similarity for inclusion.

In contrast, TM approaches using large-language-models (LLMs) might explicitly take into account predefined inclusion criteria into their article selections. Thus, LLMs might be better suited to solve the underlying problem of selecting eligible studies and have a high potential to advance the (semi-)automation of MAs further. First studies, which applied LLMs to screening literature for MAs, produced promising results [23–28]. With reported recalls ranging from 48 to 76% [23–25], some LLMs demonstrated comparable or lower performance, whereas others, with recalls between 81 and 97% [26, 27], outperformed the present TM approaches in two of the three MAs. However, each of the above LLM papers used other MA datasets for their evaluations. At the same time, transparent and reproducible research remains a challenge for LLMs [13], but less so for traditional TM. Thus, a fair comparison of LLMs and TM appears to be currently impossible. More research is needed to assess the applicability of LLMs in psychology and their performance relative to the approaches examined in the current study. Currently, from the perspective of transparency and reproducibility TM still appears to be superior.

Besides the approaches’ blindness to the inclusion criteria, the present study is subject also to other limitations. Like most evaluations of TM approaches for the automation of MAs, the article selection of already published MAs was used as the ground truth. However, this approach might introduce biases since the selected papers were known beforehand [10]. Further, the ground truth datasets were not directly available, but rather needed to be reconstructed from database searches in the present study and the (unstructured) screening documentation files of the original MAs. Studies selected by the authors of the original MAs were partially absent in the reconstructions. Thus, the utilized datasets did not exactly match the datasets of the original papers, and the reconstruction might have introduced include/exclude label inaccuracy that impaired the TM performance and its results. Thus, performance estimates should be interpreted as indicative rather than definitive, especially with respect to recall and the potential time saved against the original MAs. In line with prior work using reconstructed datasets, we explicitly report these limitations, emphasize that our potential time-saved and recall estimates are defined relative to the reconstructed datasets rather than the original MAs, and highlight that the primary goal was to demonstrate the feasibility and potential of TM approaches for psychology MAs rather than to reproduce exact screening outcomes. Fully annotated benchmark MA datasets were only recently made publicly available and were utilized for evaluating the free ASReview software [29]. Future work on TM approaches might benefit from using these benchmark datasets for better comparison between approaches.

Future studies should also try to find optimized parameter configurations to increase the recall of the various TM approaches. However, focusing solely on recall ignores that individual studies potentially can have relevant effects on the overall outcome of an MA (e.g., summary effect estimate). Thus, evaluations should also take into account individual studies’ effects on the outcome of the MA, in addition to questions over recall [30]. To increase the generalizability of the present results, the TM approaches should be evaluated on MAs from a broader variety of topics and journals – especially taking into account publicly available datasets (e.g., SYNERGY dataset [29]). Further adaptations could be examined and evaluated, too. For instance, Pham et al. [10] could be adapted to output probabilities instead of class labels. Thus, the outputs of both TM approaches could be directly compared while using the same thresholds for assigning class labels. Yet, a comparison between Pham et al. [10] and Popoff et al. [11] might be ambiguous since they were created for different purposes. While Popoff et al. [11] was written for an MA update scenario and can only be used if an MA with already screened articles exists as a training dataset, Pham et al. [10] can be used to create an entirely new MA. Hence, the starting point of the research team determines which approach ought to be used, regardless of their performance in metrics. Finally, while our findings may be applicable to systematic reviews as well, we did not investigate the performance of these approaches on systematic reviews, as this was outside the scope of our study. This should be followed-up in future research.

## Conclusion

This study applied two TM approaches [10, 11] aiming at automating study selection for MAs in three subfields of psychology, re-using publicly available codes. The results suggest that both TM approaches perform comparatively well on MAs in psychology, though neither approach captured all eligible articles. On top, with Popoff’s et al. [11] TM approach requiring pre-existing information on inclusion into the training dataset, despite its good performance, it cannot be recommended for literature screening of new MAs but only for MA updates.

While TM application may save time and workload, potentially incomplete or biased study selections may impact the quality of MAs. Hence, implications of the results of the present study are compatible with prior recommendations of using TM approaches as an aid to human screeners, second screeners, or to prioritize articles that are more likely to be included in the MA for more time-efficient manual screening [4]. In addition, future research is needed to further explore recall-maximizing parameter settings. Being based on only three datasets, and the wide performance metrics reported here, suggests that generalizability must still be established in future work with additional MAs from diverse subfields. In conclusion, TM approaches remain promising in increasing efficiency of human researchers literature screening – potentially saving time and workload for the larger research community managing an ever-growing wealth of literature.

## Supplementary Information

Supplementary Material 1.

## Data Availability

All code and the datasets generated and/or analysed during the current study are available in the OSF repository, https://osf.io/kb9mn/

## Abbreviations

MA: Meta-analysis
TM: Text-mining
DFM: Document-feature-matrix
JIF: Journal impact factor
LLM: Large-language-model
