# Supplementary material for: Automation of literature screening for meta-analyses in the field of psychology: an evaluation of two text-mining approaches
Source: Syst Rev. 2026 Aug 5;15:284. doi: 10.1186/s13643-026-03275-x (PMC13725997; doi:10.1186/s13643-026-03275-x)
Supplement: Supplementary file 1 [file 13643_2026_3275_MOESM1_ESM.pdf]

Supplementary materials

The computations and applications of the TM approaches were performed in the programming language R on a machine equipped with two Intel 4214R processors, two NVIDIA A100 / 40GB, 640 GB RAM, and running Ubuntu 20.04.6 LTS (GNU/Linux 5.13.0-40-generic x86 64).

Input files

In the present study single excel or RDS files contained all the articles which were passed into the TM approaches. For application with Pham et al. [1], the articles dataset was transformed into a table (data-frame) structure with five columns containing the available information (id, title, abstract, text, status) and each row depicting a single article. The id of the article was its initial row number, text contained the merged title and abstract, and status showed the original decision whether to include or exclude it in the MA. For Popoff et al. [2], the data-frame had three columns (ID, Abstract, In Ex). Once again, the ID was the article's initial row number, Abstract was the merged title and abstract, and In Ex showed the original decision whether to include or exclude the article in the MA. Since Pham et al. [1] used the text column with the merged title and abstract for TM, to ensure better comparison, in Popoff et al. [2] both the title and abstract were merged before being passed to the TM approach, too.

Notes on script adaptations

During application of the TM approaches technical issues appeared. For Pham et al. [1] an archived version of DMrW package had to be loaded, since the CRAN repository did not support it anymore. Furthermore, the older Text2Vec package version used in the original code files depreciated, which necessitated to update the code parts to obtain the relaxed word mover distance. Finally, it was necessary to convert all texts in the dataset to UTF-8. Likewise, Popoff et al. [2] used the 1.8.5 version of the plyr package, which depreciated but was loaded from the archive to run the code file without error. Moreover, they used a customized function convert

count(), which was not provided in the attached code version of the paper. These code parts were removed, since they were not necessary for training the support vector machine model and the guessed version of the function led to loss of information due to reducing the inversely weighted frequency counts to binary values in the DFM.

Results with confidence intervals

For the results on the meta-analyses of Everaert et al. [3], Agostini and van Zomeren [4], and Roberts et al. [5] (in this order), see Tables 1-3.

Table 1 Performance of Pham et al. [10] and Popoff et al. [11] in the Replicated Meta-Analysis of Everaert et al. [3].

		Popoff et al. [11]	
Metric	Pham et al. [10]	.8[a] threshold	.6[b] threshold
Classification Metrics			
Recall	.725 [.721, .728]	.625 [.618, .632]	.761 [.749, .773]
Accuracy	.871 [.870, .872]	.922 [.921, .923]	.904 [.902, .906]
Specificity	.873 [.872, .875]	.927 [.926, .928]	.906 [.904, .909]
Precision	.087 [.086, .087]	.124 [.122, .125]	.119 [.118, .121]
F1	.155 [.154, .156]	.207 [.204, .209]	.206 [.204, .209]
Article Screening Metrics			
Screened Articles	1123.200 [1111.791, 1134.609]	677.300 [668.472, 686.128]	859.000 [839.468, 878.532]
Screened	.137 [.135, .138]	.082 [.081, .083]	.104 [.102, .107]
Saved time	.863 [.862, .865]	.918 [.917, .919]	.896 [.893, .898]
Found Includes	97.100 [96.634, 97.566]	83.700 [82.771, 84.629]	102.000 [100.418, 103.582]
Includes Training	78.500 [77.899, 79.101]	78.500 [77.899, 79.101]	78.500 [77.899, 79.101]
Includes Approach	18.600 [17.727, 19.473]	5.200 [4.555, 5.845]	23.500 [22.263, 24.737]
Includes in Original	134.000 [134.000, 134.000]	134.000 [134.000, 134.000]	134.000 [134.000, 134.000]
Missed Includes	36.900 [36.434, 37.366]	50.300 [49.371, 51.229]	32.000 [30.418, 33.582]
Missed in %	.275 [.272, .279]	.375 [.368, .382]	.239 [.227, .251]

Note. Numbers are averages 10 runs with 5 draws of random seed studies. All values in the classification metrics section have a $SD \leq .035$. For article screening metrics, *SD*s are in parentheses. In brackets the 95% confidence intervals are shown, where the first (second) number is the lower (upper) limit. NA = Not Applicable.

[a] Articles with probability threshold > .8 selected for inclusion.

[b] Articles with probability threshold > .6 selected for inclusion.

Table 2 Performance of Pham et al. [10] and Popoff et al. [11] in the Replicated Meta-Analysis of Agostini and van Zomeren [4].

		Popoff et al. [11]	
Metric	Pham et al. [10]	.8[a] threshold	.6[b] threshold
**Classification Metrics**			
Recall	.788 [.785, .791]	.666 [.661, .671]	.805 [.798, .812]
Accuracy	.845 [.843, .846]	.920 [.919, .920]	.913 [.912, .914]
Specificity	.846 [.845, .848]	.926 [.926, .927]	.916 [.915, .917]
Precision	.119 [.119, .120]	.193 [.192, .194]	.201 [.201, .202]
F1	.207 [.206, .208]	.299 [.298, .300]	.322 [.322, .323]
**Article Screening Metrics**			
Screened Articles	1263.600 [1250.917, 1276.283]	660.100 [653.692, 666.508]	763.400 [755.566, 771.234]
Screened	.170 [.168, .172]	.089 [.088, .090]	.103 [.102, .104]
Saved time	.830 [.828, .832]	.911 [.910, .912]	.897 [.896, .898]
Found Includes	150.500 [149.885, 151.115]	127.200 [126.184, 128.216]	153.700 [152.403, 154.997]
Includes Training	111.300 [110.340, 112.260]	111.300 [110.340, 112.260]	111.300 [110.340, 112.260]
Includes Approach	39.200 [38.088, 40.312]	15.900 [15.335, 16.465]	42.400 [41.562, 43.238]
Includes in Original	191	191	191
Missed Includes	40.500 [39.885, 41.115]	63.800 [62.784, 64.816]	37.300 [36.003, 38.597]
Missed in %	.212 [0.209, .215]	.334 [.329, .339]	.195 [.188, .202]

Note. Numbers are averages 10 runs with 5 draws of random seed studies. All values in the classification metrics section have a *SD* ≤ .035. For article screening metrics, *SD*s are in parentheses. In brackets the 95% confidence intervals are shown, where the first (second) number is the lower (upper) limit. NA = Not Applicable.

[a] Articles with probability threshold > .8 selected for inclusion.

[b] Articles with probability threshold > .6 selected for inclusion.

Table 3 Performance of Pham et al. [10] and Popoff et al. [11] in the Replicated Meta-Analysis of Roberts et al. [5].

Metric	Pham et al. [10]	Popoff et al. [11] .8[a] threshold	Popoff et al. [11] .6[b] threshold
Classification Metrics			
Recall	.993 [.993, .993]	.955 [.954, .957]	.963 [.962, .965]
Accuracy	.642 [.641, .642]	.682 [.681, .682]	.682 [.682, .683]
Specificity	.601 [.601, .601]	.650 [.649, .651]	.650 [.649, .650]
Precision	.224 [.224, .224]	.240 [.240, .241]	.242 [.241, .242]
F1	.365 [.365, .365]	.384 [.384, .384]	.386 [.386, .387]
Article Screening Metrics			
Screened Articles	616.600 [616.240, 616.960]	552.200 [551.135, 553.265]	554.100 [553.147, 555.053]
Screened	.461 [.461, .461]	.413 [.412, .414]	.414 [.413, .415]
Saved time	.539 [.539, .539]	.587 [.586, .588]	.586 [.585, .587]
Found Includes	138.000 [138.000, 138.000]	132.800 [132.598, 133.002]	133.900 [133.728, 134.072]
Includes Training	132.800 [132.598, 133.002]	132.800 [132.598, 133.002]	132.800 [132.598, 133.002]
Includes Approach	5.200 [4.998, 5.402]	.000 [.000, .000]	1.100 [.928, 1.272]
Includes in Original	139	139	139
Missed Includes	1.000 [1.000, 1.000]	6.200 [5.998, 6.402]	5.100 [4.928, 5.272]
Missed in %	.007 [.007, .007]	.045 [.043, .046]	.037 [.035, .038]

Note. Numbers are averages 10 runs with 5 draws of random seed studies. All values in the classification metrics section have a *SD* ≤ .035. For article screening metrics, *SD*s are in parentheses. In brackets the 95% confidence intervals are shown, where the first (second) number is the lower (upper) limit. NA = Not Applicable.

[a] Articles with probability threshold > .8 selected for inclusion.

[b] Articles with probability threshold > .6 selected for inclusion.

Sensitivity Analysis for potentially saved time

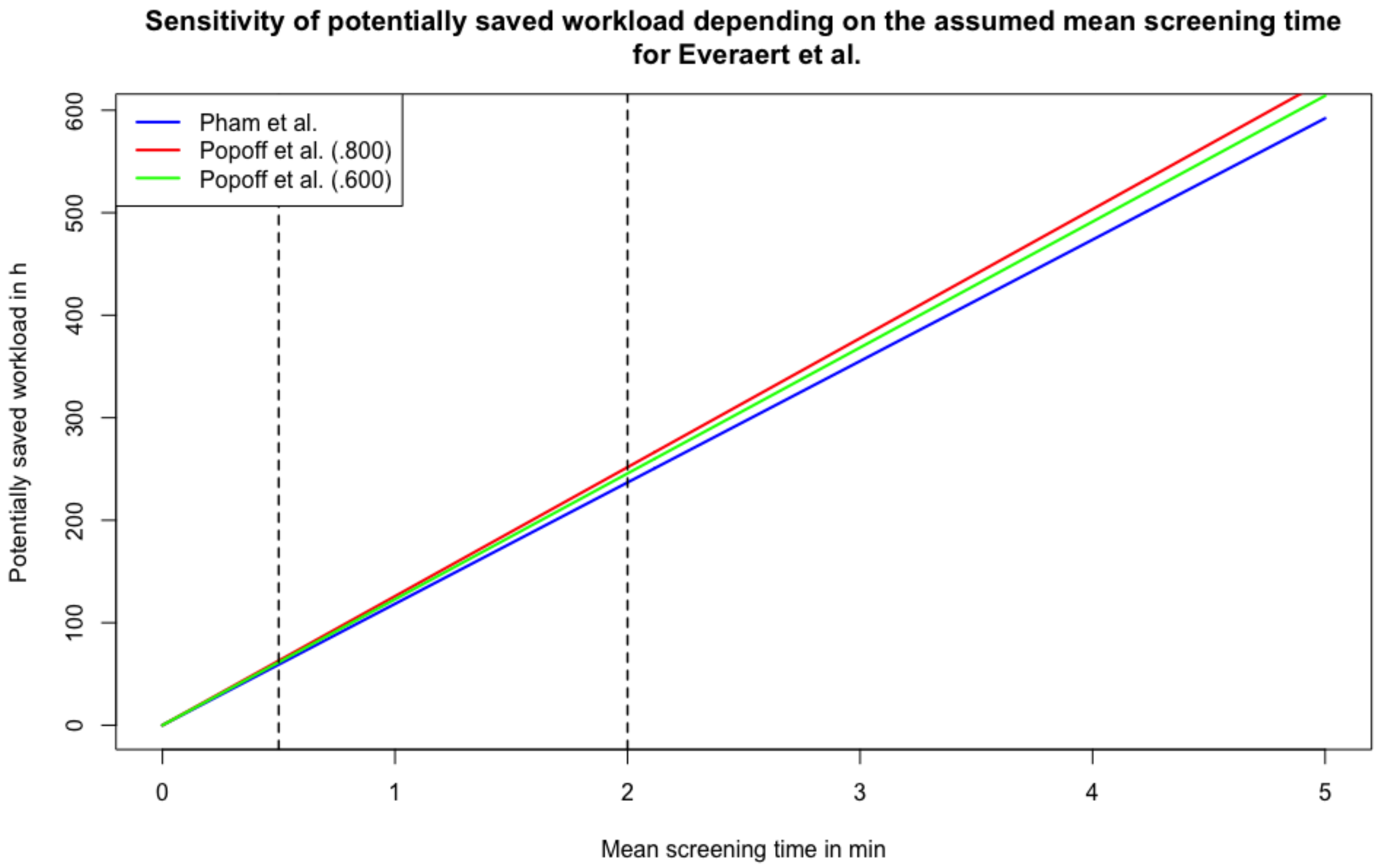

Fig. 1 Illustration of the potential time saved when applying different average screening time assumptions for Everaert et al. [3]. The dotted lines indicate the 30 s or 2 min boundaries used in our paper.

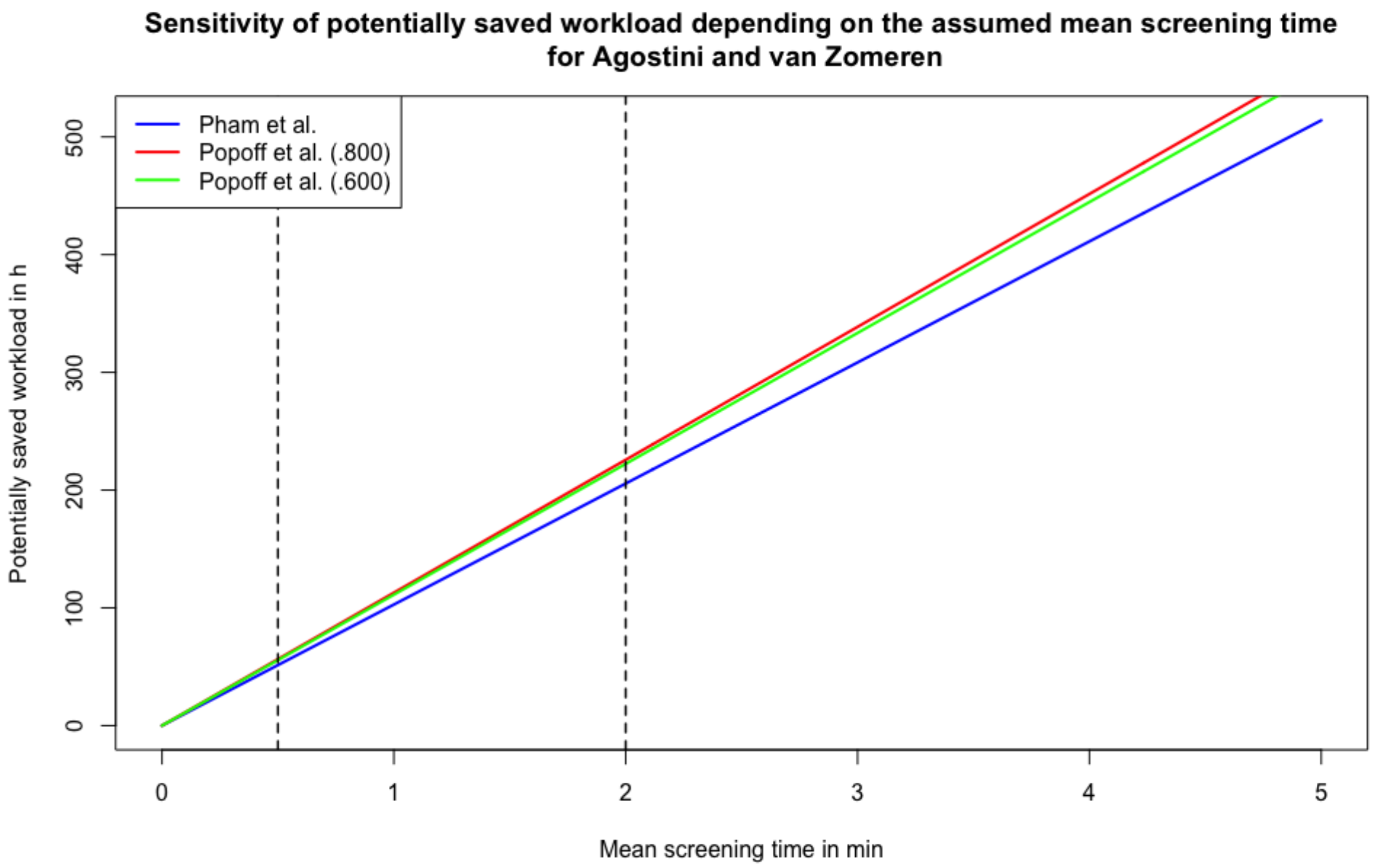

Fig. 2 Illustration of the potential time saved when applying different average screening time assumptions Agostini and van Zomeren [4]. The dotted lines indicate the 30 s or 2 min boundaries used in our paper.

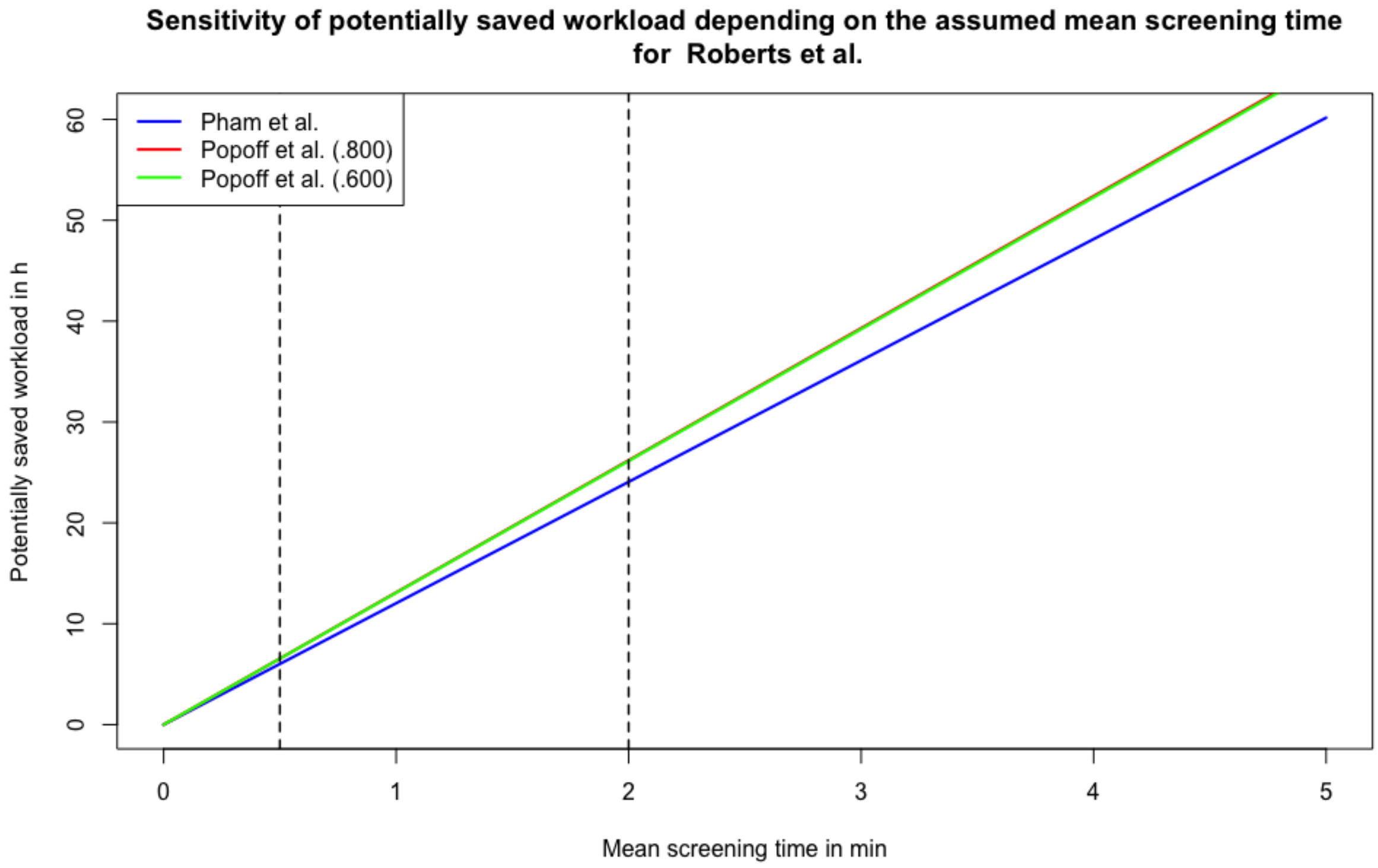

Fig. 3 Illustration of the potential time saved when applying different average screening time assumptions for Roberts et al. [5]. The dotted lines indicate the 30 s or 2 min boundaries used in our paper.

References

[1] Pham B, Jovanovic J, Bagheri E, Antony J, Ashoor H, Nguyen TT, et al. Text mining to support abstract screening for knowledge syntheses: a semi-automated workflow. Syst Rev. 2021; doi:10.1186/s13643-021-01700-x.

[2] Popoff E, Besada M, Jansen J, Cope S, Kanters S. Aligning text mining and machine learning algorithms with best practices for study selection in systematic literature reviews. Syst Rev. 2020; doi:10.1186/s13643-020-01520-5.

[3] Everaert J, Vrijsen JN, Martin-Willett R, van de Kraats L, Joormann J. A meta-analytic review of the relationship between explicit memory bias and depression: depression features an explicit memory bias that persists beyond a depressive episode. Psychol Bull. 2022;148:435-63

[4] Agostini M, van Zomeren M. Toward a comprehensive and potentially cross-cultural model of why people engage in collective action: a quantitative research synthesis of four motivations and structural constraints. Psychol Bull. 2021;147:667-700.

[5] Roberts BRT, MacLeod CM, Fernandes MA. The enactment effect: a systematic review and meta-analysis of behavioral, neuroimaging, and patient studies. Psychol Bull. 2022;148:397-434.
